# Enzymatic Degradation of Polyethylene Terephthalate Model Substrates by Esterase E4

**DOI:** 10.3390/biology15070540

**Published:** 2026-03-27

**Authors:** Shuyan Duan, Huifang Yang, Rumeng Sun, Jiankang Ma, Kun Wang

**Affiliations:** College of Food Science and Pharmaceutical Engineering, Zaozhuang University, Zaozhuang 277160, China

**Keywords:** E4, FASTase, PET, BHET, enzymatic activity

## Abstract

As the demand for polyethylene terephthalate (PET) continues to rise, significant environmental pollution caused by challenges in PET degradation has garnered global attention. Given the crucial role of esterases in depolymerizing PET into reusable monomers, such enzymes capable of degrading plastics have attracted considerable interest. In this study, we used the previously reported ultra-efficient mutant of the PET-degrading enzyme *Ideonella sakaiensis* PETase, known as FASTase, as a positive control. We investigated the PET-degrading activity of esterase E4, derived from *Altererythrobacter indicus*. The results demonstrated that E4 exhibits degradative activity toward the PET substrate bis(2-hydroxyethyl) terephthalate, the PET model substrate bis(benzyloxyethyl) terephthalate, and PET nanoparticles. Notably, E4 retains its degradation activity under high-temperature and high-salt conditions and can enhance the enzymatic activity of FASTase when acting synergistically. Given the low structural and sequence similarity between E4 and *Is*PETase, our research broadens the scope for screening PET-degrading enzymes.

## Simple Summary

The actual contribution of PET-degrading enzymes to the degradation of PET pollutants in the environment is directly linked to their practical feasibility. As demand for polyethylene terephthalate (PET) continues to rise, significant environmental pollution from PET degradation has garnered global attention. Given the essential role of esterases in breaking down PET into reusable monomers, enzymes with the ability to degrade plastics have garnered significant attention. In this study, we employed a previously described highly efficient variant of *Ideonella sakaiensis* PETase, known as FASTase, as a positive control. We evaluated the PET-degrading capacity of esterase E4, which was originally identified from *Altererythrobacter indicus*. The results indicated that E4 exhibits hydrolytic activity toward bis(2-hydroxyethyl) terephthalate and degrades the PET model substrates bis(benzyloxyethyl) terephthalate (3PET) and PET nanoparticles, demonstrating better thermal stability than the highly efficient mutant FASTase. The low structural and sequence similarity between E4 and *Is*PETase not only highlights its uniqueness but also significantly expands the scope for future screening of PET-degrading enzymes.

## 1. Introduction

Plastic products possess excellent physical and chemical properties, making them indispensable in various aspects of human life. However, the dramatic increase in global plastic production and consumption has led to the accumulation of plastic waste in the environment. By 2050, the total amount of plastic waste accumulated worldwide is projected to reach 33 billion tonnes [1]. Discarded plastic waste significantly contributes to environmental issues, including marine and soil pollution. Plastics can decompose into microplastic particles smaller than 5 mm, which are often ingested by marine organisms, accumulate in their bodies, and ultimately transfer to humans who consume these organisms [2]. In 2022, Leslie et al. reported for the first time that microplastics have been detected in human blood [3].

The primary chemical bond in PET plastic is the ester bond, which can be hydrolyzed by PET-degrading enzymes to facilitate PET degradation. PET exists in two forms: crystalline and disordered amorphous states [4]. The crystalline structure of PET is too stable for enzymes to degrade it effectively. However, when heated to around 65 °C, PET begins to soften and transitions to a disordered amorphous state, allowing enzymes to penetrate the material and interact with the ester bonds. At this temperature, however, the enzymes gradually lose their activity [4,5]. Consequently, a significant area of research in modifying PET-degrading enzymes focuses on enhancing their thermal stability, with much of the effort directed toward *Ideonella sakaiensis* (*Is*PETase).

In 2016, Yoshida et al. identified a new bacterial species, *Ideonella sakaiensis*, which degrades PET by secreting *Is*PETase and mono(2-hydroxyethyl) terephthalate hydrolase (MHETase) [6]. *Is*PETase hydrolyzes PET to produce mono(2-hydroxyethyl) terephthalate (MHET), terephthalic acid (TPA), bis(2-hydroxyethyl) terephthalate (BHET), and ethylene glycol (EG) [7]. These components are ultimately hydrolyzed by *Is*PETase and its coenzyme, MHETase, into TPA and EG (Figure S1) [8,9]. *Is*PETase has a melting temperature of only about 40 °C, which limits its thermal stability at elevated temperatures. Furthermore, *Is*PETase demonstrates strong catalytic activity, primarily for short-chain fatty acid esters, with its catalytic efficiency gradually decreasing as the chain length increases [6]. Based on structural information and computer-aided enzyme modification, several highly efficient, thermally stable mutants, such as *Is*PETase^TM^ [10], DuraPETase [11], DepoPETase [12], FASTase [13], and HotPETase [14], have been reported.

The acquisition of these highly efficient mutants establishes a robust foundation for industrial applications. However, the modifications have primarily focused on enhancing activity and thermal stability, without adequately addressing other potential conditions, such as those related to plastic pollutants (e.g., solvents and salts) [15]. Meanwhile, global plastic consumption continues to rise rapidly, and relying solely on *Is*PETase as the primary reference sequence for enzyme modification and exploration is inadequate to tackle the growing and complex issue of environmental plastic pollution. Consequently, there is an urgent need to broaden the screening reference sequences for PET-degrading enzymes to identify enzymes capable of withstanding diverse environmental conditions.

Based on whole-genome sequence analysis [16], Li et al. identified a novel member of the esterase family, E4, from the rhizobacterium *Altererythrobacter indicus* DSM 18604T [17,18]. They conducted studies on its protein structure and enzymatic properties [18]. E4 exhibits vigorous catalytic activity for para-nitrophenyl esters with fatty acid chain lengths ranging from C2 to C14. It demonstrates high thermal stability, showing almost no activity loss after being heated at 70 °C for one hour and retaining 20–40% activity after heating at 100 °C for one hour. Additionally, E4 displays significant salt tolerance, maintaining over 61% activity at high concentrations of NaCl, and shows high resistance to organic solvents [18]. These findings suggest that E4 is a promising biocatalyst for ester bond formation that can withstand various extreme environmental conditions. However, further advances in the practical application of E4 were not achieved in this study.

**Figure 1:**
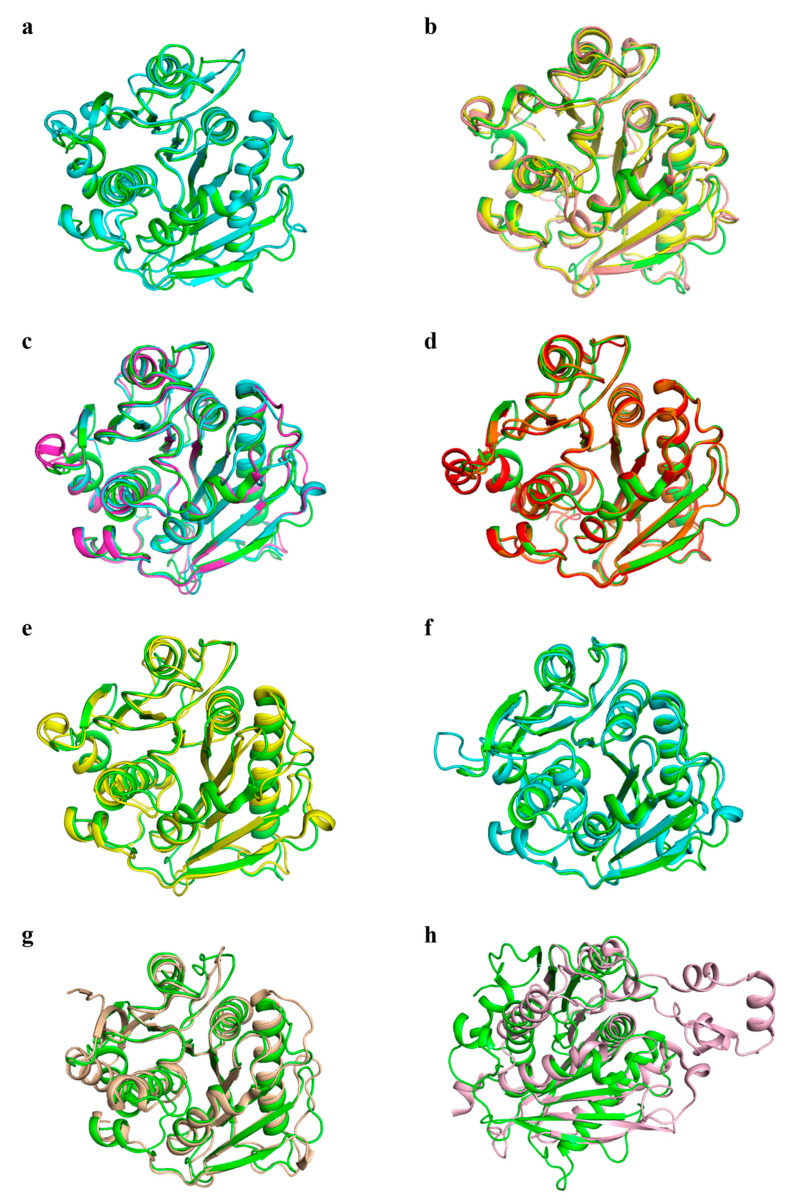
(**a**) Structural alignment between *Is*PETase (PDB: 5XJH, green) and PET6 (PDB: 7Z6B, blue) yields an RMSD of 0.48 Å. (**b**) Structural alignment among *Is*PETase (PDB: 5XJH, green), PLE628 (PDB: 7VMD, yellow; RMSD: 0.804 Å), and PLE629 (PDB: 7VPA, magenta; RMSD: 0.565 Å). (**c**) Structural alignment among *Is*PETase (PDB: 5XJH, green), PE-H (PDB: 6SBN, blue; RMSD: 0.671 Å), and *Pbauz*Cut (PDB: 8AIT, purple; RMSD: 0.637 Å). (**d**) Structural alignment among *Is*PETase (PDB: 5XJH, green), *Rg*PETase (PDB: 7DZT, red; RMSD: 0.271 Å), and *Rg*Cut-II (PDB: 8AIR, purple; RMSD: 0.366 Å). (**e**) Structural alignment between *Is*PETase (PDB: 5XJH, green) and *Ps*Cut (PDB: 8AIS, yellow) yields an RMSD of 0.707 Å. (**f**) Structural alignment between *Is*PETase (PDB: 5XJH, green) and Est119 (PDB: 6AID, yellow) yields an RMSD of 0.614 Å. (**g**) Structural alignment between *Is*PETase (PDB: 5XJH, green) and PET30 (PDB: 7PZJ, deep yellow) yields an RMSD of 0.728 Å. (**h**) Structural alignment between *Is*PETase (PDB: 5XJH, green) and PET46 (PDB: 8B4U, light pink) yields an RMSD of 1.471 Å.

**Figure 2:**
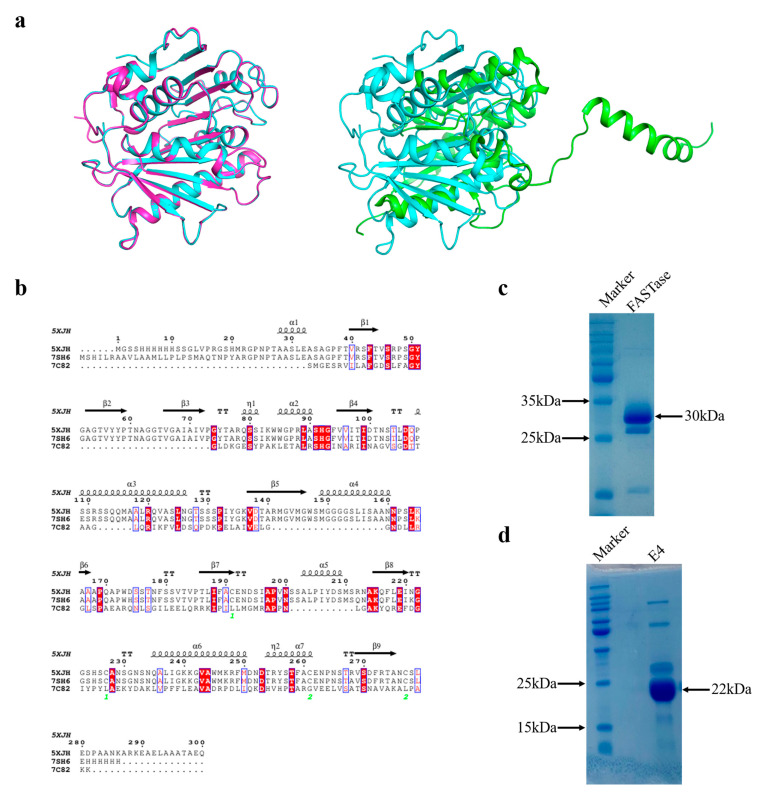
(**a**) Structural alignment of *Is*PETase (PDB: 5XJH, blue), FASTase (PDB: 7SH6, magenta), and E4 (PDB: 7C82, green). Red background with white text indicates that the amino acids in this column are identical across all sequences. (**b**) Sequence alignment of *Is*PETase (PDB: 5XJH), FASTase (PDB: 7SH6), and E4 (PDB: 7C82). (**c**) SDS-PAGE analysis of FASTase. (**d**) SDS-PAGE analysis of E4.

Given that both *Is*PETase and E4 are esterases, and considering the structural similarity of E4’s substrate, para-nitrophenyl ester(pNP), to the PET degradation intermediates BHET and MHET (Figure S2), we hypothesized that E4 may possess PET-degrading activity and conducted relevant experimental validations. The experimental results indicate that E4 exhibits hydrolytic activity toward BHET and degrades the PET model substrate, bis(benzyloxyethyl) terephthalate (3PET), as well as PET nanoparticles, demonstrating better thermal stability than the highly efficient mutant FASTase. E4 shows low sequence and structural similarity to *Is*PETase (Figure 1 and Figure 2). Our work lays a solid foundation for subsequent structural and molecular mechanism studies on E4’s degradation of PET, thereby expanding the reference sequences for screening PET-degrading enzymes.

## 2. Materials and Methods

### 2.1. Strains, Plasmids, and Materials

*Escherichia coli* (*E. coli*) SHuffle T7 competent cells were obtained from WEIDI (Shanghai, China). The coding sequence of FASTase was modified by removing the N-terminal signal peptide (amino acids 1–27). The coding sequences of FASTase and E4 were cloned into the pET-22b(+) vector, which was synthesized by Sangon Biotech (Shanghai, China). BHET was purchased from Sigma (St. Louis, MO, USA), and PET nanoparticles were obtained from ZHONGKEKEYOU (Beijing, China). The synthesis of bis(benzyloxyethyl) terephthalate (3PET) was carried out in our collaborative laboratory (Green Pharmaceutical Synthesis Laboratory, Zaozhuang University). Bromophenol blue and phenol red were sourced from Macklin (Shanghai, China). The bacterial active protein extraction reagent was purchased from Beyotime (Shanghai, China).

### 2.2. Protein Expression and Purification

*E. coli* SHuffle T7 cells harboring pET-22b-FASTase or pET-22b-E4 were cultured in 50 mL of Luria–Bertani medium supplemented with ampicillin (100 μg/mL) at 37 °C with shaking at 180 rpm overnight. A volume of 15 mL of the overnight culture was transferred into 200 mL of fresh Luria–Bertani medium containing ampicillin and incubated at 37 °C in a shaker at 220 rpm until the optical density at 600 nm (OD600) measured using a spectrophotometer obtained from UNICO (Shanghai, China) reached approximately 0.8. The culture was then cooled to 18 °C, and 0.4 mM Isopropyl β-d-thiogalactopyranoside was added to induce target protein expression. The culture continued to grow at 18 °C for 20 h at 180 rpm.

Cells were harvested by centrifugation at 3500× *g* for 30 min at 4 °C, and the supernatant was discarded. The cells were lysed using a bacterial protein extraction reagent, and the cellular debris was removed by centrifugation at 10,000× *g* for 60 min at 4 °C. The supernatant was subsequently applied to a nickel-affinity column that had been pre-equilibrated with equilibration buffer (50 mM Tris HCl, pH 8.0; 500 mM NaCl; 10 mM imidazole, pH 8.0; and 5% glycerol). The target protein was eluted using an elution buffer consisting of 50 mM Tris-HCl, pH 8.0; 500 mM NaCl; and 250 mM imidazole, pH 8.0, supplemented with 5% glycerol [19]. The eluted fraction was concentrated to approximately 1 mL using ultrafiltration tubes, and the protein buffer was exchanged to buffer A (20 mM Tris HCl, pH 7.4; 100 mM NaCl; and 2 mM DTT). The purity of the purified proteins was assessed by 12.5% SDS-PAGE. Subsequently, FASTase and E4 were adjusted to the same protein concentration using buffer A and stored at −80 °C for future use.

### 2.3. Evaluation of the Hydrolytic Activity, High-Salt Tolerance, and Thermostability of Enzyme E4

A volume of 100 mL of protein buffer solution containing 20 mM Tris-HCl (pH 7.4) and 100 mM NaCl was mixed with 1.5 g of agar. The mixture was heated in a microwave until the agar was completely dissolved. Next, 4 mL of a 100 mM BHET solution (dissolved in DMSO) was added, thoroughly mixed, and then poured onto a plate to a thickness of approximately 20 mm. Once solidified, the BHET activity assay plate was obtained. Wells were manually punched into the plate for subsequent use. Using FASTase as a positive control, the hydrolytic activity and thermal stability of E4 were evaluated, with a protein buffer solution serving as the negative control. Equal amounts of FASTase, E4, and the buffer solution were added to the agar plates, and the changes in the size of the hydrolysis zone were monitored systematically over 12 days. Following this initial observation, both FASTase and E4 were subjected to thermal treatment at 50 °C, 60 °C, 70 °C, 80 °C, 90 °C, and 100 °C for 2 h. Subsequently, BHET solid agar plates were used to assess whether FASTase and E4 retained their hydrolytic activity toward BHET. Again, equal amounts of FASTase, E4, and the buffer solution were applied to the agar plates, and changes in the hydrolysis zone size were monitored for an additional 9 days.

Using FASTase and protein buffer solution as the positive and negative controls, respectively, a colorimetric method was employed to quantitatively analyze the hydrolytic activity of E4 on BHET, as optimized by Jessica Lusty Beech et al. [20]. The enzyme activity detection system contained 300 nM enzyme, 1 mM BHET, 0.1 mM bromophenol blue or phenol red, 10 mM CaCl_2_, 10% DMSO, and 5 mM BES (pH 7.0, for bromophenol blue) or HEPES (pH 8.0, for phenol red). Absorbance changes at 550 nm (for phenol red) or at 615 nm (for bromophenol blue) were continuously monitored for 18 h, with measurements taken every 15 min. All experiments were performed in triplicate.

After heat treatment of FASTase and E4 at 50 °C, 60 °C, 70 °C, 80 °C, 90 °C, and 100 °C for 2 h, the same colorimetric method was used to quantitatively assess the thermal stability of E4 in hydrolyzing BHET. The enzyme activity detection system consisted of 300 nM heat-treated enzyme, 1 mM BHET, 0.1 mM bromophenol blue or phenol red, 10 mM CaCl_2_, 10% DMSO, and 5 mM BES (pH 7.0, for bromophenol blue) or HEPES (pH 8.0, for phenol red). Absorbance change at 550 nm (for phenol red) or at 615 nm (for bromophenol blue) was continuously monitored over 18 h, with measurements recorded every 15 min. All experiments were performed in triplicate.

#### 2.3.1. Determination of E4 Hydrolytic Activity on BHET Under High-Salt Conditions

The colorimetric method was employed for the quantitative analysis of E4 hydrolytic activity toward BHET under high-salt conditions. The enzyme activity detection system contained 300 nM enzyme, 1 mM BHET, 0.1 mM bromothymol blue or phenol red, 10 mM CaCl_2_, 10% DMSO, and 5 mM BES (pH 7.0, for bromophenol blue) or HEPES (pH 8.0, for phenol red). Distilled water (0 M NaCl) and NaCl solutions of 0.5 M, 1 M, 2 M, 3 M, 4 M, and 5 M were added to the reaction system to achieve a total volume of 200 µL for the enzyme activity assay. Absorbance at 550 nm (for phenol red) or at 615 nm (for bromophenol blue) was continuously monitored for 18 h, with measurements taken every 15 min. All experiments were performed in triplicate.

#### 2.3.2. E4 Hydrolytic Activity Toward 3PET

The 3PET hydrolysis activity assay for E4 was optimized based on the work of Jessica Lusty Beech et al. [20]. Twenty milligrams of synthesized 3PET was dissolved in 600 µL of acetone, and the solution was gradually added to pre-warmed 50 mM Tris-HCl buffer (pH 8.0). After the system stabilized, the mixture was centrifuged, and the supernatant was collected to obtain a 3PET emulsion for subsequent use. FASTase and protein buffer solution served as positive and negative controls, respectively, to quantitatively evaluate the hydrolytic activity of E4 toward 3PET using a colorimetric method. Ten microliters of enzyme was added to 190 µL of the prepared 3PET emulsion. Absorbance at 580 nm was continuously monitored for 4 h, with optical density (OD 580 nm) readings recorded every 15 min. All experiments were performed in triplicate.

#### 2.3.3. E4 Hydrolytic Activity Toward PET Nanoparticles

The assay for E4 hydrolysis activity on PET nanoparticles was optimized based on the method described by Valentina Pirillo et al. [21]. A colorimetric approach was used to analyze the activity of E4 in hydrolyzing PET nanoparticles. For the enzyme activity detection system, 2 µL of PET nanoparticles (25 mg/mL) were used, and four experimental groups were set up with enzyme volumes of 20 µL, 10 µL, 5 µL, and 0 µL, respectively. A buffer solution containing 20 mM Tris-HCl (pH 7.4) and 100 mM NaCl was added to adjust the total reaction volume to 200 µL. After 5 days, absorbance at 600 nm was measured. All experiments were performed in triplicate.

## 3. Results

### 3.1. E4 Exhibits Low Sequence and Structural Similarity to IsPETase

The PAZy database compiles data on the activities, genes, and proteins of validated plastic-degrading enzymes that target both fossil-fuel-based polymers and those primarily derived from renewable resources [22]. Among the enzymes capable of degrading such polymers, there are 125 PET-degrading enzymes, including cutinase (EC 3.1.1.74), lipases (EC 3.1.1.3), and carboxylesterases (EC 3.1.1.1/EC 3.1.1.101/EC 3.1.1.2). Our summary analysis of these 125 enzymes showed that 62 possessed EC numbers, of which 26 belonged to the carboxylesterase family (Table 1). Among these 26 members, 25 were bacterial esterases, one was an archaeal esterase, and none were of eukaryotic origin (Table 1).

Of the 26 carboxylesterases, eleven for which protein structures are available (Table 1, Figure 1) were selected to be compared with *Is*PETase based on structural similarity. All eleven proteins exhibited a high degree of structural resemblance to *Is*PETase (Figure 1). The PETase from *Rhizobacter gummiphilus* NS21 showed the highest similarity, with a root-mean-square deviation (RMSD) of 0.271 Å. At the same time, PET46 from the *Candidatus Bathyarchaeota* archaeon exhibited the lowest similarity, with an RMSD of 1.471 Å.

Esterase E4 and *Is*PETase both exhibit hydrolytic activity on the model substrate p-nitrophenyl ester. Given that the structure of p-nitrophenyl butyrate (pNPB) closely resembles that of BHET (as shown in Figure S2), it is hypothesized that E4 may also hydrolyze BHET. To test this hypothesis, we first performed a bioinformatics analysis of E4. Structural comparison between E4 and the ultra-efficient *Is*PETase variant FASTase (Figure 2a) revealed low similarity, with an RMSD value exceeding 10 Å. Moreover, sequence alignment showed no significant similarity (Figure 2b). To further examine the potential PET-degrading activity of E4, we purified both high-purity E4 and FASTase using nickel affinity chromatography. The theoretical molecular weight of E4 is 22 kDa, while that of FASTase is 30 kDa (Figure 2c,d).

### 3.2. E4 Demonstrates the Capability to Degrade BHET

**Figure 3:**
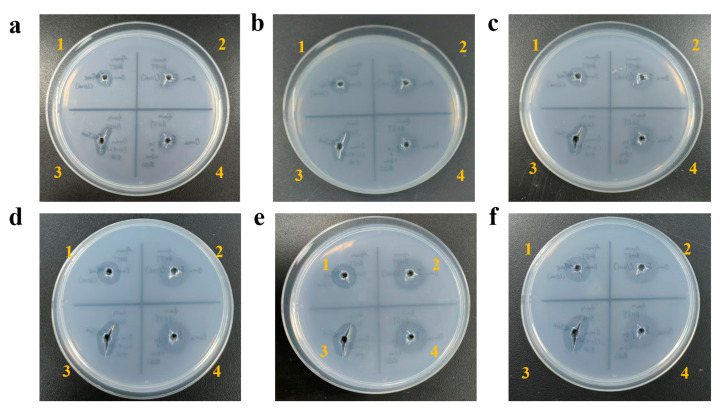
Panels (**a**–**f**) sequentially show the hydrolysis zones after incubating with E4 and FASTase (control) for 1, 2, 3, 6, 8, and 12 days, respectively, during the observation of E4 activity toward BHET hydrolysis. Here, “1” indicates FASTase, “2” indicates E4, and “3” and “4” indicate FASTase and E4, respectively, with 10% glycerol added to the enzyme system.

Using FASTase as a positive control, a BHET hydrolysis halo assay indicated that E4 possesses BHET-hydrolyzing activity. Continuous monitoring of enzyme activity over 1, 2, 3, 6, 8, and 12 days showed that the hydrolysis halo gradually enlarged from day 1 to day 6, with a relatively rapid increase in size (Figure 3a–d). After day 6, the rate of halo expansion slowed noticeably (Figure 3e,f). Li et al. reported that the addition of 10% glycerol could enhance the pNPB-hydrolyzing activity of E4 by more than twofold [18]. We therefore examined whether adding 10% glycerol would similarly affect the BHET hydrolysis activity of E4 and FASTase. The halo assay revealed that glycerol did not significantly enhance the BHET-hydrolyzing activity of E4 (Figure 3a–f).

### 3.3. E4 Exhibits Superior Thermal Stability Compared to FASTase

**Figure 4:**
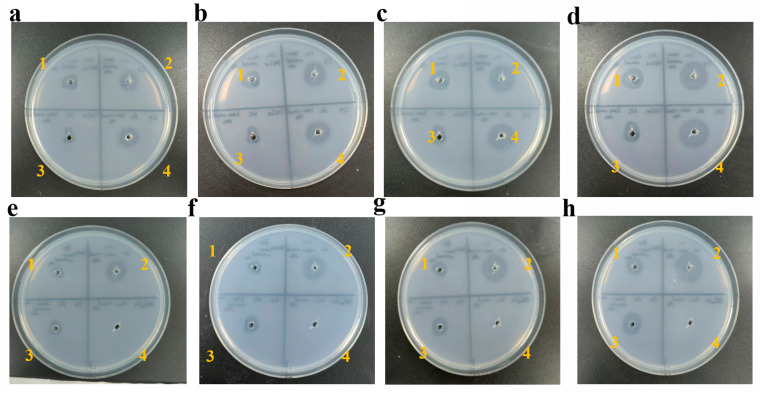
Panels (**a**–**d**) show the hydrolysis zone of BHET after treatment at 50 °C (samples 1 and 2) and 60 °C (samples 3 and 4) for 2 h, observed on days 2, 3, 5, and 9 (samples 1 and 3: FASTase; samples 2 and 4: E4). Panels (**e**–**h**) show the hydrolysis zone of BHET after treatment at 70 °C (samples 1 and 2) and 80 °C (samples 3 and 4) for 2 h, also observed on days 2, 3, 5, and 9 (samples 1 and 4: FASTase; samples 2 and 3: E4).

The BHET hydrolysis assay was performed after heat treatment at 50 °C, 60 °C, 70 °C, 80 °C, 90 °C, and 100 °C for 2 h. The activity of E4 and FASTase was measured, and changes in hydrolysis were evaluated on days 2, 3, 5, and 9 post-treatment. The results showed no significant difference in activity between E4 and FASTase after treatment at 50 °C (Figure 4a–d). In contrast, under heat treatments from 60 °C to 90 °C, E4 exhibited markedly higher thermal stability than FASTase (Figure 4e–h, Figure S3). E4 retained its BHET-hydrolyzing activity even after 2 h at 90 °C (Figure S3), whereas FASTase was almost completely inactivated following 2 h at 70 °C (Figure 4e).

**Figure 5:**
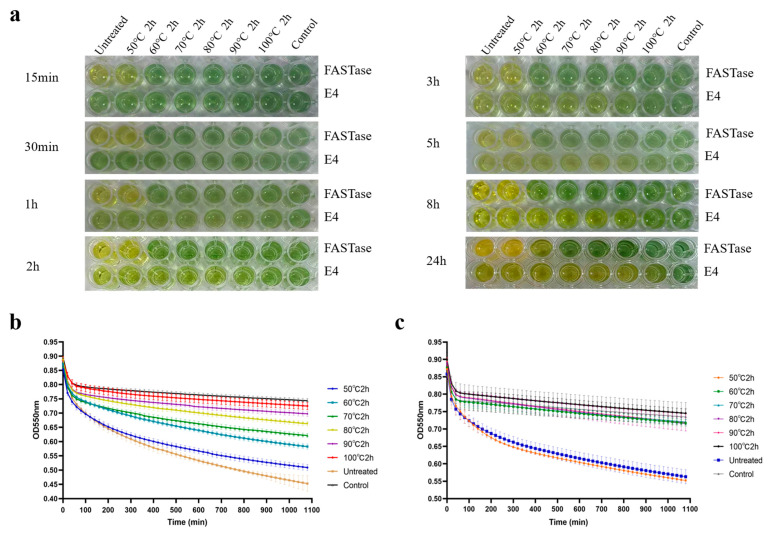
Enzyme activity and thermal stability assays. (**a**) Assay using bromothymol blue as the indicator, with FASTase as a control, to detect the activity and thermal stability of E4. (**b**) Assay based on absorbance change at 550 nm using phenol red as the indicator to evaluate the activity and thermal stability of E4. (**c**) Assay under the same conditions as in (**b**) to evaluate the activity and thermal stability of FASTase.

Using bromothymol blue as an indicator to assess the thermal stability of E4 during BHET hydrolysis, in this assay, green indicates no reaction, yellow-green a partial reaction, and yellow a complete reaction. Unheated enzymes served as the positive control, while a group without added protein samples was used as the negative control. Enzyme activity changes in E4 and FASTase were measured after heat treatment at 50 °C, 60 °C, 70 °C, 80 °C, 90 °C, and 100 °C for 2 h. As shown in Figure 5a, FASTase initially exhibited higher activity than E4; FASTase showed partial BHET hydrolysis after 15 min, whereas E4 began to display partial hydrolysis only after 2 h. However, FASTase was significantly less thermally stable than E4.

After treatment at 100 °C, E4 could still hydrolyze a small amount of BHET after 24 h, consistent with the halo assay results (Figure 4e–h). In contrast, FASTase was inactivated after heat treatment at 70 °C for 2 h (Figure 5a). Using phenol red as an indicator and FASTase as a positive control, absorbance at 550 nm was monitored continuously for 18 h to quantify the thermal stability of E4 during BHET hydrolysis (Figure 5b,c). In this assay, a greater change in OD before and after the reaction indicates higher enzyme thermal stability. The activity of FASTase declined rapidly with increasing temperature, with no significant loss observed only after 2 h at 50 °C; almost complete loss of activity was observed at other treatment temperatures (60 °C, 70 °C, 80 °C, 90 °C, and 100 °C) (Figure 5c). In contrast, results from the bromothymol blue assay (Figure 5a) were consistent with those from the phenol red assay, confirming that the activity of E4 gradually decreased with increasing temperature and that it retained partial activity even after 2 h at 90 °C (Figure 5b).

### 3.4. E4 Exhibits Lower Activity than FASTase in Degrading BHET and Shows a Synergistic Effect When Combined with FASTase

**Figure 6:**
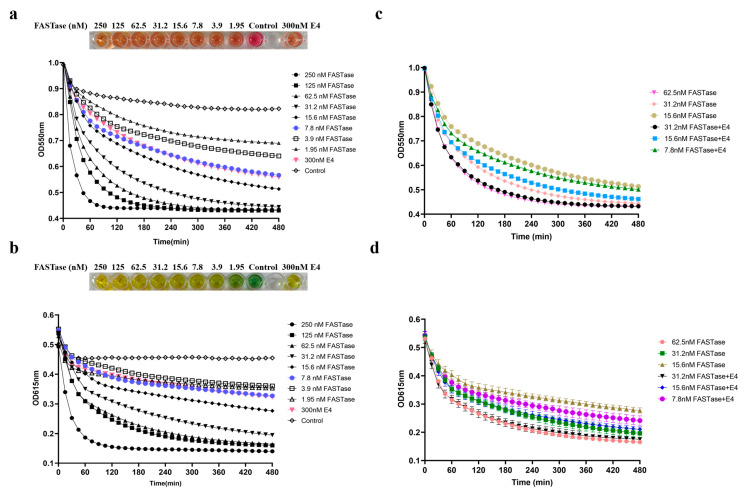
Enzymatic degradation of BHET assessed with different indicators. (**a**) Comparison of the degradation activity of E4 and FASTase using phenol red. (**b**) Comparison of the degradation activity of E4 and FASTase using bromothymol blue. (**c**) Investigation of the synergistic effect of E4 and FASTase using phenol red. (**d**) Investigation of the synergistic effect of E4 and FASTase using bromothymol blue.

The colorimetric assay was used to quantitatively evaluate and compare the activities of FASTase and E4. In this method, a larger change in OD value indicates higher enzyme activity. Detection was carried out using two reagents, bromothymol blue (OD 615 nm) and phenol red (OD 550 nm), by monitoring color changes and corresponding OD variations at the respective wavelengths. The results showed that FASTase activity was significantly higher than that of E4. Both detection reagents, bromothymol blue and phenol red, indicated that 7.8 nM FASTase exhibited activity approximately equivalent to that of 300 nM E4 (Figure 6a,b), suggesting that FASTase is about 40 times more active than E4.

To assess whether E4 could act synergistically with FASTase, 300 nM E4 was added to reaction systems containing 31.2 nM, 15.6 nM, and 7.8 nM FASTase, respectively. The results indicated that these systems achieved activity levels comparable to those containing only 62.5 nM, 31.2 nM, and 15.6 nM FASTase (Figure 6c,d), suggesting that E4 can work synergistically with FASTase to enhance its effectiveness.

### 3.5. E4 Retains BHET-Hydrolyzing Activity Even Under High-Salt Conditions

**Figure 7:**
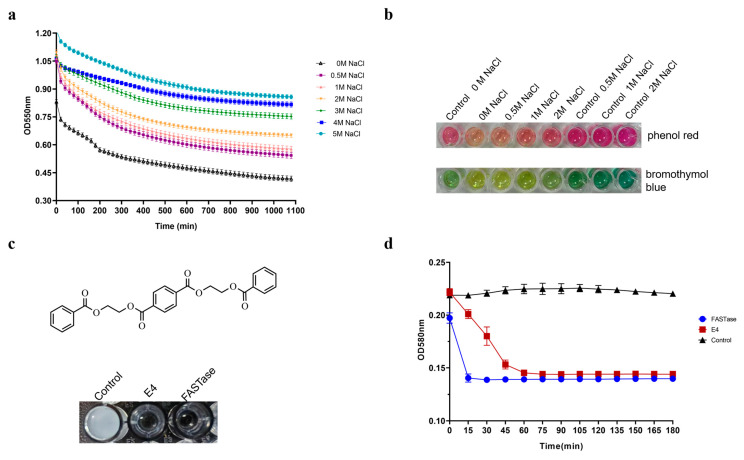
(**a**) Detection of E4’s high salt tolerance by measuring absorbance changes at 550 nm using phenol red as an indicator. (**b**) Assessment of enzyme activity changes under high salt conditions for E4 by monitoring color variations with phenol red and bromothymol blue as indicators. (**c**) Chemical structure of 3PET and evaluation of E4’s degradation activity on a 3PET emulsion. (**d**) Detection of E4’s degradation activity on the 3PET emulsion, using FASTase as a positive control, by measuring absorbance changes at 580 nm.

To determine whether E4 retains its BHET-hydrolyzing activity under high-salt conditions, we measured changes in E4 activity by adding NaCl at concentrations of 0 M, 0.5 M, 1 M, 2 M, 3 M, 4 M, and 5 M to the reaction system. Phenol red was used as the detection reagent to monitor absorbance at 550 nm. As shown in Figure 7a, the activity of E4 gradually decreased with increasing salt concentration.

E4 maintained high activity at NaCl concentrations of 0.5 M, 1 M, and 2 M. Furthermore, activity was evaluated using both phenol red and bromothymol blue under the same reaction conditions (0 M, 0.5 M, 1 M, and 2 M NaCl). With phenol red, a lighter purple color corresponds to higher activity, whereas with bromothymol blue, a lighter green color indicates stronger activity. The results presented in Figure 7b are consistent with the colorimetric data in Figure 7a, confirming that activity progressively declines with increasing salt concentration. At the same time, E4 continues to show strong activity at 0.5 M, 1 M, and 2 M NaCl.

### 3.6. E4 Exhibits Degradation Activity for the PET Mode Substrate 3PET

Bis(benzyloxyethyl) terephthalate (3PET, Figure 7c) is a short-chain derivative of PET and is commonly used as a model substrate to evaluate the PET-degrading activity of enzymes. A 3PET emulsion was added to a 96-well plate, followed by the addition of equal volumes of buffer, E4, and FASTase to the reaction system. Absorbance changes were continuously monitored at a wavelength of 580 nm over a period of 3 h.

As shown in Figure 7d, the reaction began immediately upon the addition of FASTase to the 3PET emulsion, resulting in complete degradation within 15 min. E4 also exhibited activity against 3PET; however, its degradation rate was significantly slower than that of FASTase, reaching an OD comparable to FASTase’s only after approximately 90 min of reaction. The corresponding changes in turbidity indicated that the control group remained milky white, while the reaction groups containing FASTase and E4 became clear (Figure 7c).

### 3.7. E4 Exhibits the Ability to Degrade PET Nanoparticles

Using FASTase as a positive control and an enzyme-free group as a negative control, 20 µL, 10 µL, and 5 µL of E4 were added to 50 µg of PET nanoparticles. After incubation at 37 °C for 5 days, the absorbance was measured. In this assay, a decrease in absorbance indicates PET nanoparticle degradation by the enzyme, with a greater decrease corresponding to higher enzymatic activity.

**Figure 8:**
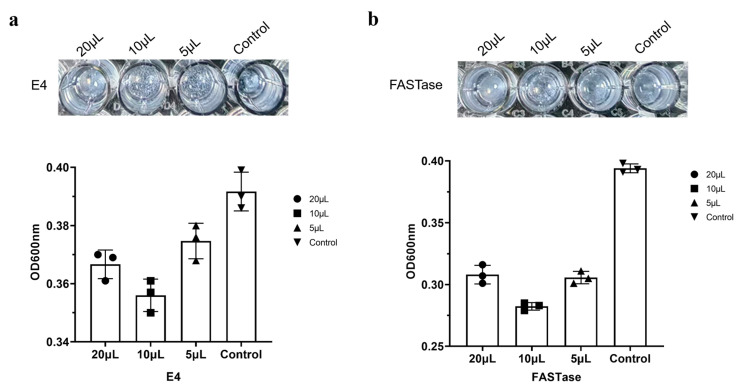
(**a**) Degradation activity of E4 on PET nanoparticles monitored by measuring the absorbance change at 600 nm. (**b**) Degradation activity of FASTase on PET nanoparticles monitored by measuring the absorbance change at 600 nm.

As shown in Figure 8a, E4 is capable of degrading PET nanoparticles, with the highest degradation efficiency observed at an enzyme volume of 10 µL. Enzyme concentration inhibition was evident at 20 µL. This trend in degradation is similar to that of FASTase; however, the degradation activity of E4 is lower than that of FASTase, as presented in Figure 8b.

## 4. Discussion

The practical contribution of PET-degrading enzymes to the degradation of PET pollutants in the environment directly determines their practical feasibility. Current research on PET-degrading enzymes often uses IsPETase as the reference sequence, primarily focusing on developing thermostable and efficient IsPETase mutants, whereas relatively little attention is paid to other environmental conditions associated with plastic pollution. There remains a need for adaptive engineering of PET-degrading enzymes for other application scenarios and conditions, such as solvent and salt stability, and for improved activity against higher-crystallinity PET [15,23,24]. Additionally, the discovery of PET-degrading enzymes often adopts an evolutionary perspective, relying on high sequence or structural similarity to IsPETase as screening criteria (see Figure 1 and Table 1). This approach confines the selection of reference sequences for PET-degrading enzymes to a limited sequence space.

To broaden the reference sequences for screening PET-degrading enzymes, this study first investigated the chemical similarity of the substrates acted upon by relevant enzymes and their enzymatic properties, aiming to explore the degradation effect of esterase E4 (which exhibits low sequence and structural similarity to *Is*PETase) on PET model substrates. The results showed that E4 possesses hydrolytic activity toward BHET and the PET model substrate 3PET, as well as degradation activity toward PET nanoparticles. Although the activity of E4 in degrading PET model substrates was lower than that of FASTase, its thermostability was significantly better (Figure 5).

Although the degradation rate of this enzyme was relatively low compared with that of FASTase, this finding provides a foundation for subsequent optimization. Similar to *Is*PETase, despite its initially weak enzymatic activity [6], numerous efficient mutants, including *Is*PETase™ [10], FASTase [13], DuraPETase [11], DepoPETase [12], and HotPETase [14], have been identified through enzyme engineering since its plastic-degrading capability was recognized. This paper reports the degradation activity of E4 against PET model substrates, laying the groundwork for further analysis of the E4-ET model substrate complex and for related enzyme molecular engineering efforts. Through molecular docking studies of E4 with BHET, we identified amino acids in E4 that exhibit polar interactions with BHET, namely Ser13, Tyr18, and Tyr26 (Figure S4). We anticipate obtaining the crystal structure of the E4-BHET complex to facilitate a detailed elucidation of its reaction mechanism.

Currently, there is an urgent need for PET-degrading enzymes that remain effective under high-salt conditions [15]; however, reports on the impact of high salt on the activity of plastic-degrading enzymes are scarce. This study demonstrates that E4 maintains strong activity under high-salt conditions. Hydrolysis zone experiments indicated that the expansion rate of the hydrolysis zone for E4 decreased significantly after six days of reaction. This observation aligns with previously reported findings for FASTase, suggesting that continuous supplementation of fresh enzyme is crucial for sustaining activity [25]. According to existing reports, *Is*PETase exhibits concentration-dependent inhibition affecting its activity [6,25]; similarly, E4 also displayed concentration-dependent inhibition during the degradation of PET nanoparticles (Figure 8). Given that the concentration inhibition of *Is*PETase can be alleviated through enzyme engineering [25], we speculate that subsequent engineering of E4 may also eliminate this inhibition.

## 5. Conclusions

In summary, we present a novel perspective for expanding the screening of PET-degrading enzyme sequences, along with innovative methods for identifying degrading enzymes for various types of plastics. We anticipate identifying additional plastic-degrading enzymes through comparative studies of their enzymatic properties.

## Figures and Tables

**Table 1:** The 26 currently available PET carboxylesterases.

Microbial Host/Enzyme/Gene	EC Number	PDB Entry	GenBank/UniProt/MGnify
Bacteria			
Pseudomonadota			
*Ideonella sakaiensis* 201-F6, *Is*PETase, *ISF6_4831* (DuraPETase)(ThermoPETase) (Fast-PETase)(HotPETase) (DepoPETase)	EC 3.1.1.101	5XJH, and others	A0A0K8P6T7
*Oleispira antarctica RB-8*, *PET5*, *LipA* (=*Oacut*)	EC 3.1.1.1		R4YKL9_OLEAN
*Vibrio gazogenes*, *PET6*, *BSQ33_03270*	EC 3.1.1.1	7Z6B	A0A1Z2SIQ1_VIBGA
*Caldimonas brevitalea*, *PET12* (*PbCut*; *SbCut*), *AAW51_2473*	EC 3.1.1.1		A0A0G3BI90_9BURK
*Marinobacter* sp., *PLE628*	EC 3.1.1.1	7VMD	OK558825
*Marinobacter* sp., *PLE629*	EC 3.1.1.1	7VPA	OK558824
*Halopseudomonas aestusnigri VGXO14*, *PE-H*, *B7O88_11480*	EC 3.1.1.1	6SBN, and others	A0A1H6AD45
*Pseudomonas saudimassiliensis*, *PsCut*	EC 3.1.1.1	8AIS	A0A078MGG8
*Pseudomonas bauzanensis*, *PbauzCut*	EC 3.1.1.1	8AIT	A0A031MKR8
*Pseudomonas alcaligenes*, *PaCut*	EC 3.1.1.1		SUD16364.1
*Pseudomonas pseudoalcaligenes*, *PpEst* (*tesA*)	EC 3.1.1.2		W6R2Y2
*Pseudomonas* sp., *esterase MG8*	EC 3.1.1.1		MGYP000532440779
*Pseudomonas* sp., *strain 9.2*, *EstB*	EC 3.1.1.1		WP_085690612
*Rhizobacter gummiphilus NS21*, *RgPETase/RgCut-I*	EC 3.1.1.1	7DZT, and others	A4W93_05950
*Rhizobacter gummiphilus*, *RgCut-II*	EC 3.1.1.1	8AIR	WP_085749238.1
*Acidovorax delafieldii*, *AdCut*	EC 3.1.1.1		Q8RR62
Actinomycetota			
*T. fusca*, *TfCut_1* (*Cut-1.kw3*)	3.1.1.101		E5BBQ2
*T. halotolerans*, *Thh_Est*	EC 3.1.1.1		H6WX58
*T. alba* (*AHK119*), *Est1* (*Hydrolase 4*); *Enzyme 708*	EC 3.1.1.1		BAI99230
*T. alba AHK119*, *Est119*, *est2*	EC 3.1.1.1	6AID	F7IX06
Bacillota			
*Bacillus subtilis 4P3-11*, *BsEstB*	EC 3.1.1.1		ADH43200.1
*Caldibacillus thermoamylovorans*, *Ces19_14*	EC 3.1.1.1		WP_034767800.1
*Caldibacillus thermoamylovorans*, *Ces39_5*	EC 3.1.1.1		WP_108898647.1
Bacteroidota			
*Aequorivita* sp. *CIP111184*, *PET27*	EC 3.1.1.1		WP_111881932
*Kaistella* (*Chryseobacterium*) *jeonii*, *PET30*	EC 3.1.1.1	7PZJ	WP_039353427
Archaea			
*Candidatus Bathyarchaeota archaeon*, *PET46*	EC 3.1.1.73	8B4U	RLI42440.1

## Data Availability

The original contributions presented in this study are included in the article/Supplementary Materials. Further inquiries can be directed to the corresponding author.

## Supplementary Materials

The following supporting information can be downloaded at: https://www.mdpi.com/article/10.3390/biology15070540/s1. Figure S1: Schematic diagram illustrating the enzymatic degradation of PET by IsPETase and MHETase. Figure S2: Comparison of the Chemical Structures of p-Nitrophenyl Butyrate, BHET, and MHET. Figure S3: The changes in the hydrolysis region of BHET after treatment at 90 °C and 100 °C for 2 h. Figure S4: The docking result of BHET with enzyme E4.
