# Supplementary material for: Enzymatic Degradation of Polyethylene Terephthalate Model Substrates by Esterase E4
Source: Biology (Basel). 2026 Mar 27;15(7):540. doi: 10.3390/biology15070540 (PMC13072011; doi:10.3390/biology15070540)
Supplement: Supplementary file 1 [file biology-15-00540-s001.zip › biology-4182155-supplementary.pdf]

Supporting Information

Figure S1

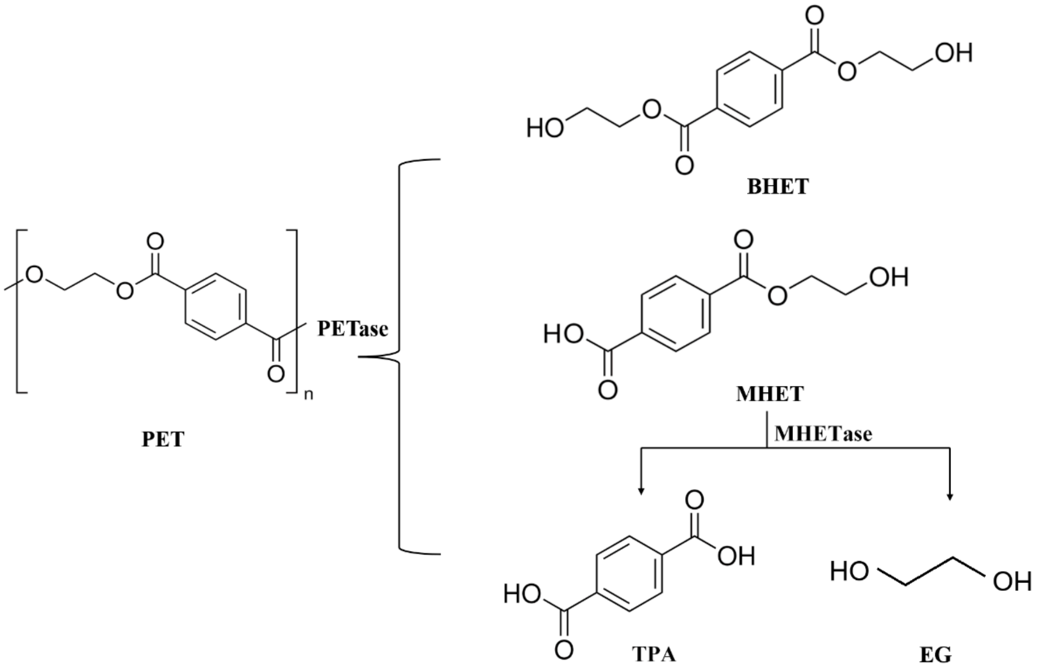

Figure S1. Schematic diagram illustrating the enzymatic degradation of PET by *Is*PETase and MHETase.

Figure S2

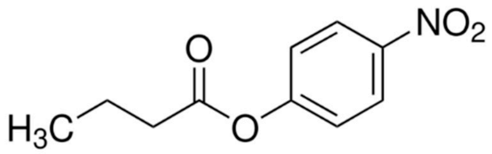

P-nitrophenyl butyrate

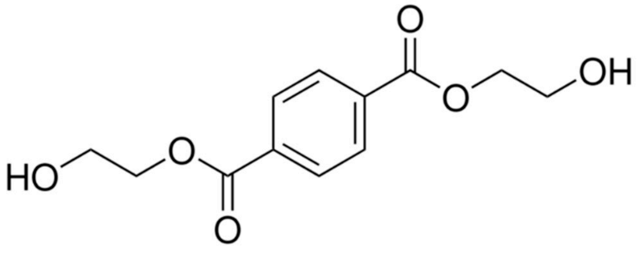

BHET

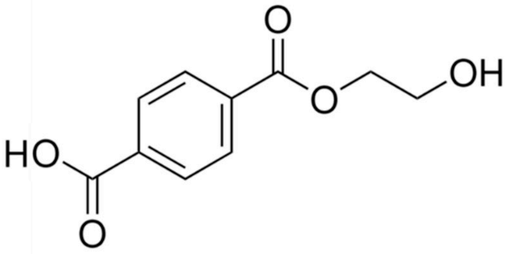

MHET

Figure S2. Comparison of the Chemical Structures of p-Nitrophenyl Butyrate, BHET, and MHET.

Figure S3

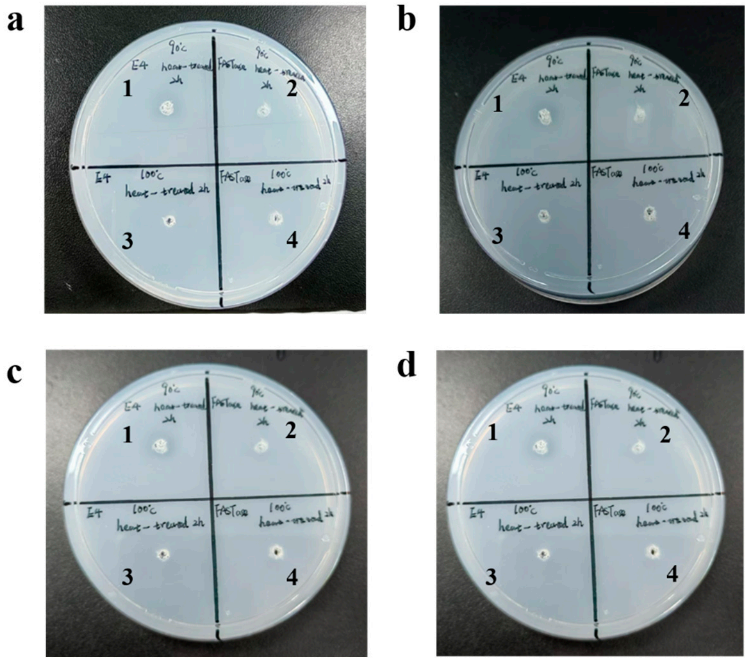

Figure S3. Panels (a)-(d) illustrate the changes in the hydrolysis ring of BHET over time (observed on days 2, 3, 5, and 9) after treatment at 90°C (samples 1 & 2) and 100°C (samples 3 & 4) for 2 hours. Samples 1 and 3 correspond to enzyme E4, while samples 2 and 4 correspond to enzyme FASTase.

Figure S4

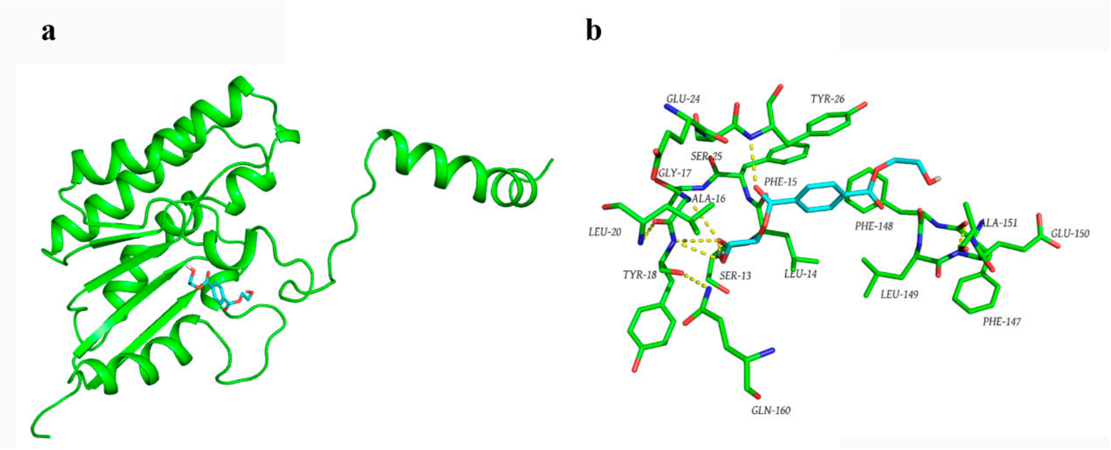

Figure S4. (a) Molecular docking model of BHET bound to enzyme E4. (b) Analysis of the polar interactions between BHET and protein amino acid residues within a 4 Å radius.
